# Structural Insights into HPV16 E6:E6AP:p53 Complex Formation and Inhibition by Covalent Peptides

**DOI:** 10.1063/4.0000978

**Published:** 2025-10-27

**Authors:** Aaron H Nile

**Affiliations:** Calico Life Sciences LLC, South San Francisco, CA, USA

## Abstract

Human papillomavirus (HPV) is the primary driver of cervical, head and neck, and anal cancers through its ability to hijack the cellular E3-ligase E6AP and promote degradation of the tumor suppressor protein p53. This mechanism, mediated by the HPV E6 protein, has been recognized for decades but has remained difficult to therapeutically target.

We recently determined the high-resolution (∼3.3 Å) cryo-electron microscopy structure of the full-length HPV16 E6:E6AP:p53 complex, uncovering an unexpectedly high affinity interaction and large surface interface between HPV16 E6 and E6AP, far beyond the canonical E6AP LXXLL motif. This provided new structural insights and identified critical, previously unknown interfaces essential for complex stability.

In parallel, we developed peptide discovery strategies, including Reversibly Reactive Affinity Selection–Mass Spectrometry (ReAct-ASMS) coupled with rational design of covalent peptide inhibitors or "reactides". Through these studies we identified and optimized covalent peptides targeting a critical cysteine residue within HPV16 E6, effectively disrupting its interaction with E6AP.

Together, our integrated structural, computational, and chemical biology approaches provide a deeper understanding of HPV-driven carcinogenesis, and importantly, establish a foundation for the development of selective covalent targeted strategies to inhibit HPV-driven cancers.

